# Societal cost of oropharyngeal cancer by human papillomavirus status, cancer stage, and subsite

**DOI:** 10.1371/journal.pone.0220534

**Published:** 2019-07-29

**Authors:** Maria Silfverschiöld, Johanna Sjövall, Johan Wennerberg, Ellinor Östensson, Lennart Greiff

**Affiliations:** 1 Department of Otorhinolaryngology, Head & Neck Surgery, Skåne University Hospital, Lund, Sweden; 2 Department of Clinical Sciences, Lund University, Lund, Sweden; 3 Department of Medical Epidemiology & Biostatistics, Karolinska Institute, Stockholm, Sweden; 4 Department of Children’s & Women’s Health, Karolinska Hospital, Stockholm, Sweden; Chang Gung Memorial Hospital at Linkou, TAIWAN

## Abstract

**Background:**

The incidence of oropharyngeal cancer (OPC) is increasing, particularly human papillomavirus (HPV)-associated OPC. The aim of this study was to specify the total societal cost of OPC by HPV status, cancer stage, and subsite using a bottom-up cost-of-illness approach.

**Methods:**

We analyzed 121 consecutive patients with OPC from the Southern Health Care Region of Sweden. We estimated the direct medical costs and indirect costs (e.g., disease-related morbidity and premature death) from 1 month prior to OPC diagnosis until 3 years after treatment completion.

**Results:**

The mean total cost per patient was €103 386 for HPV-positive and €120 244 for HPV-negative OPC. Eighty-one percent of the patients analyzed were HPV-positive: Accordingly, HPV-positive OPC represented 79% of the total cost of OPC. The mean total cost of stage I, II, III, IVA, IVB, and IVC, regardless of HPV status, was €59 424, €57 000, €69 246, €115 770, €234 459, and €21 930, respectively, of which indirect costs were estimated at €22 493 (37.8%), €14 754 (25.9%), €28 681 (41.4%), €67 107 (58%), €166 280 (70.9%), and €0. Tonsillar cancer represented 64% of OPC, with a mean total cost of €117 512 per patient.

**Conclusion:**

The societal cost of OPC is substantial. HPV-associated OPC comprises 79% of the total cost of this disease. The data presented in this study may be used in analytical models to aid decision makers in determining the potential value of gender-neutral HPV vaccination.

## Introduction

Head and neck cancer (HNC) is the 6^th^ most prevalent cancer worldwide [1]. The incidence of oropharyngeal cancer (OPC), a subset of HNC localized to the tonsils, the base of the tongue, and the soft palate/pharyngeal wall, is increasing, particularly human papillomavirus (HPV)-associated OPC [2]. HPV-positive OPC has been linked to male gender, younger age, and white race [3]. HPV-positive OPC is associated with less advanced primary cancer lesions, but with a higher likelihood of regional metastasis at presentation [3]. Overall, HPV-positive OPC has a better prognosis than HPV-negative OPC [4].

The increase in the incidence of OPC makes studies of its societal cost important. Detailed information on HPV-positive OPC is particularly called for due to the increasing incidence of this condition and the possibility of primary prevention through HPV vaccination. However, specific costs are also needed for each clinical stage of OPC, as disease progression may lead to altered costs, which is relevant to health care programs that aim to achieve early (i.e., early-stage) detection and treatment. Finally, specific costs by OPC subsite, i.e., tonsils, base of the tongue, and soft palate/pharyngeal wall, are also necessary, as changes to current treatment protocols may be subsite-specific; for example, the introduction of transoral robotic-assisted surgery for resectable tonsillar cancer.

A series of studies have described the costs of OPC and other HNCs [5–9]. However, these reports do not include indirect costs associated with morbidity, sick leave, etc. Moreover, they include data obtained using top-down approaches that look at group-level information on HPV-status, cancer stage, etc., not bottom-up approaches that look at individual-level information. In a previous report using a top-down approach, our research team estimated that the societal economic burden of HPV-associated precancers and cancers (using prevalence data from the literature) was €94 million in 2006, of which OPC represented €12 million (12.8%) [9]. Taken together, the information on the societal cost of OPC is still incomplete.

In this population-based study of patients with OPC in Southern Sweden, we aimed, for the first time, to specify the total societal cost, direct medical costs, and indirect costs of OPC by HPV status, cancer stage, and subsite using a bottom-up cost-of-illness approach.

## Material and methods

### Study design

The study had a prevalence-based cost-of-illness design, which is a methodology commonly used to investigate the economic burden of diseases [10]. All identifiable, individual-level, direct medical (i.e., work-up, treatment, hospitalization, etc.) and indirect (i.e., sick leave, premature death, etc.) costs linked to OPC as a primary diagnosis were included in the estimate. Costs were stratified by HPV status, cancer stage (according to the TNM Classification of Malignant Tumour, 7^th^ ed., Wiley-Blackwell, Hoboken, NJ), and OPC subsite, and expressed in Euro (€) based on 2017 values. The observation period covered approximately 1 month prior to OPC diagnosis to 3 years after treatment completion. Approval of the study protocol was obtained from the Regional Ethics Review Board, Lund, Sweden (ref. number 2017/647).

### Study population

Using the Swedish National Quality Registry for Head and Neck Cancer, we identified 121 consecutive patients from the Southern Health Care Region of Sweden who were diagnosed with OPC: tonsillar cancer (International Statistical Classification of Diseases and Related Health Problems, Revision 10, 2^nd^ edition (ICD-10) code C09) base of the tongue cancer (ICD-10 code C01), and soft palate/pharyngeal wall cancer (ICD-10 code C10) during 2011–2014 and treated at Skåne University Hospital [11].

### HPV status

A key focus of our analysis was the distribution of the costs of OPC by HPV status. Therefore, information on HPV DNA and/or p16 status was retrieved from laboratory reports. In this context, the presence of HPV DNA indicates an association between HPV and OPC, while p16 positivity is a surrogate marker for HPV positivity [12]. For the purposes of our analyses, we regarded p16 as a fully justifiable marker of HPV infection. If data were lacking, tissue samples were analyzed for HPV DNA or p16 *de novo*. Data on HPV status were eventually available for all 121 patients.

### Treatment

The patients in this study received external radiotherapy to 68 Gy to the primary tumor and to any neck node metastases. Elective radiotherapy to 54 Gy was also given to the ipsilateral neck region, and contralateral treatment was considered for patients with primary lesions extending close to or over the midline. Concomitant chemotherapy was offered to patients with advanced-stage disease; surgery was reserved for residual or recurrent disease. In 90% of the cases, the treatment intention was curative.

### Costs

#### Direct medical costs

We used a bottom-up approach to determine the OPC-specific direct medical costs associated with inpatient and outpatient work-up, treatment, and follow-up. The costs were derived from the hospitals’ financial departments and verified against medical records in order to identify OPC-specific costs only. The cost of palliative care or advanced care in a patient’s home (“Avancerad sjukvård i hemmet”) were calculated using an average cost of €748 per day for palliative care and €215 per day for advanced care in a patient’s home (data obtained from Skåne County Council) multiplied by the duration of treatment.

#### Indirect costs

Indirect costs were defined as the loss of productivity that occurred due to a patient’s inability to work (i.e., sick leave or premature death). To estimate production loss, the length of work absence was multiplied by labor costs. We estimated annual labor costs, i.e., annual income from full employment including taxes and social fees, at 31.42% based on official statistics [13]. Assuming 220 workdays per year, and considering an 8-hour work day, this amounts to 1760 working hours per year. Using the human capital method and price levels from 2016, the cost of a full work day was estimated at €235 for individuals aged 20–64 years [14]. Costs of production loss corresponding to premature death were estimated based on the number of working years lost prior to a retirement age of 65 years. The number of working years lost was calculated by identifying the diagnosis-specific cause of death, age at time of death, and the number of working years lost. Costs due to future production loss were calculated using a 3% annual discount in accordance with health economic recommendations [15].

## Results

The study population comprised 121 patients with OPC, of which 34 (28%) were females and 87 (72%) males (Table 1). The overall mean age was 62 years (range 33–83 years), with corresponding values of 61 years and 63 years among females and males, respectively. The mean age of HPV-positive and HPV-negative patients was 61 years and 67 years, respectively. Twenty-nine percent of the study population died within the 3-year follow-up period: 21.5% from OPC and 7.4% from other causes.

**Table 1:** Patient characteristics by human papillomavirus (HPV) status.

	Numbers (%)		
	Total	HPV-positive	HPV-negative
**Study population**	121	98 (81.0%)	23 (19.0%)
**Gender**			
Male	87 (71.9%)	74 (85.1%)	13 (14.9%)
Female	34 (28.1%)	24 (70.6%)	10 (29.4%)
**OPC subsite**			
Tonsillar	77 (63.6%)	68 (88.3%)	9 (11.7%)
Base of the tongue	32 (26.4%)	26 (81.3%)	6 (18.7%)
Soft palate/pharyngeal wall	12 (10.0%)	4 (33.3%)	8 (66.7%)
**T-stage**			
1	26 (21.5%)	24 (92.3%)	2 (7.7%)
2	54 (44.6%)	46 (85.2%)	8 (14.8%)
3	20 (16.5%)	17 (85%)	3 (15%)
4a	14 (11.6%)	8 (57.1%)	6 (42.9%)
4b	7 (5.8%)	3 (42.9%)	4 (57.1%)
**N-stage**			
0	29 (24%)	24 (82.8%)	5 (17.2%)
1	18 (14.9%)	13 (72.2%)	5 (27.8%)
2a	10 (8.3%)	10 (100%)	0 (0%)
2b	48 (39.7%)	42 (87.5%)	6 (12.5%)
2c	11 (9.1%)	4 (36.4%)	7 (63.6%)
3	5 (4.1%)	5 (100%)	0 (0%)
**M-stage**			
0	117 (96.7%)	95 (81.2%)	22 (18.8%)
1	4 (3.3%)	3 (75%)	1 (25%)
**Clinical stage**			
I	3 (2.5%)	2 (66.7%)	1 (33.3%)
II	14 (11.6%)	13 (92.9%)	1 (7.1%)
III	20 (16.5%)	15 (75%)	5 (25%)
IVA	70 (57.9%)	59 (84.3)	11 (15.7%)
IVB	10 (8.3%)	6 (60%)	4 (40%)
IVC	4 (3.3%)	3 (75%)	1 (25%)
**Smoking habits**			
Smokers (or recently stopped)	45 (37.1%)	29 (64.4%)	16 (35.6%)
Ex-smokers	34 (28.1%)	27 (79.4%)	7 (20.6%)
Never smoked	42 (34.7%)	42 (100%)	0 (0%)
**Initial treatment intention**			
Curative	109 (90%)	93 (85.3%)	16 (14.7%)
Palliative	12 (10%)	5 (41.7%)	7 (58.3%)
**Survival**			
Deceased during 3-year follow-up	35 (28.9%)	17 (48.6%)	18 (51.4%)
Died of OPC	26 (21.5%)	11 (42.3%)	15 (57.7%)

The mean total cost of HPV-positive OPC from 1 month prior to diagnosis to 3 years after treatment completion was €103 386 per patient (Table 2). For HPV-negative OPC, the corresponding cost was €120 244.

**Table 2:** Detailed mean direct medical costs, indirect costs, and total mean cost (in € based on 2017 values) of oropharyngeal cancer by human papillomavirus (HPV) status.

	HPV-positive	HPV-negative
**Direct medical costs**		
Inpatient	16 477	31 756
Outpatient	26 734	19 683
Palliative care	1 335	6 124
***Total direct medical costs***	**44 546**	**57 563**
**Indirect costs**		
Morbidity	42 926	10 026
Mortality (premature death)*	15 914	52 655
***Total indirect costs***	**58 840**	**62 680**
**TOTAL COST**	**103 386**	**120 244**

*Costs were discounted at 3% annually.

The mean total cost per patient of OPC, regardless of HPV status, was €106 590, with €47 020 attributable to direct medical costs, i.e., inpatient and outpatient care, and €59 570 to indirect costs, i.e., due to disease-related sick leave and premature death (Table 3). When looking at the mean direct medical costs, indirect costs, and total cost per patient by clinical stage, the cost of palliative care for stage IVC was revealed to be nil, due to the fact that costs of informal care and assistance from elderly care facilities were not available (Table 3). For the same patient group, the cost was nil for morbidity and for mortality, because all individuals were older than retirement age, and therefore not eligible for sick leave pay.

**Table 3:** Detailed mean direct medical costs, indirect costs, and total mean cost (in € based on 2017 values) of oropharyngeal cancer by clinical stage.

	I (n = 3)	II (n = 14)	III (n = 20)	IVA (n = 70)	IVB (n = 10)	IVC (n = 4)	All (n = 121)
**Direct medical costs**							
Inpatient	14 191	15 406	11 468	19 798	43 737	8 556	19 381
Outpatient	22 740	23 055	29 097	26 170	21 435	13 374	25 394
Palliative care	n/a	3 785	n/a	2 695	3 007	n/a	2 245
***Total direct medical costs***	**36 931**	**42 246**	**40 565**	**48 663**	**68 179**	**21 930**	**47 020**
**Indirect costs**							
Morbidity	22 493	14 754	23 586	41 445	79 042	n/a	36 672
Mortality (premature death)*	n/a	n/a	5 095	25 662	87 238	n/a	22 898
***Total indirect costs***	**22 493**	**14 754**	**28 681**	**67 107**	**166 280**	**n/a**	**59 570**
**TOTAL COST**	**59 424**	**57 000**	**69 246**	**115 770**	**234 459**	**21 930**	**106 590**

*Costs were discounted at 3% annually; n/a denotes “not available”.

When costs were analyzed by OPC subsite, tonsillar cancer represented the largest group, i.e., 77 (64%) of the cases, and produced a mean total cost of €117 512 (Table 4). For soft palate/pharyngeal wall cancer, the direct medical costs were higher than those for other subsites, possibly due to the relatively poor prognosis of this disease, which leads to additional work-up and treatment, and corresponding increased direct medical costs.

**Table 4:** Detailed mean direct medical costs, indirect costs, and total mean cost (in € based on 2017 values) of oropharyngeal cancer by subsite.

	Tonsillar C09 (n = 77)	Base of tongue C01 (n = 32)	SP/PhW C10 (n = 12)	All (n = 121)
**Direct medical costs**				
Inpatient	16 169	17 569	44 827	19 381
Outpatient	26 885	25 252	16 198	25 394
Palliative care	2 261	558	6 644	2 245
***Total direct medical costs***	**45 316**	**43 379**	**67 669**	**47 020**
**Indirect costs**				
Morbidity	43 274	30 226	11 501	36 672
Mortality (premature death)*	28 922	1 568	41 119	22 898
***Total indirect costs***	**72 196**	**31 794**	**52 620**	**59 570**
**TOTAL COST**	**117 512**	**75 173**	**120 289**	**106 590**

*Costs were discounted at 3% annually; SP/PhW denotes soft palate/pharyngeal wall.

The mean hospital stay was 14 days (range 0–115 days), with HPV-positive OPC having a mean stay of 10 days (range 0–72) compared to 29 days for HPV-negative OPC (range 0–115) (Table 5).

**Table 5:** Mean hospital stay (days) by subsite and human papillomavirus (HPV) status.

	Total	HPV-positive	HPV-negative
**Subsite**			
Tonsillar	10	11	9
Base of the tongue	11	8	24
Soft palate/pharyngeal wall	49	25	55
**TOTAL**	**14**	**10**	**29**

Mean cost for HPV-negative OPC was 16.3% higher than for HPV-positive OPC. Moreover, the mean total cost for male patients was less (€93 954) than that for female patients (€135 984). Among the male patients, HPV-positive OPC accounted for 87% of the total cost. Twenty-one percent of HPV-negative OPC was detected in the advanced stages IVB and IVC, compared to 9% of HPV-positive OPC (Table 1). The total cost of OPC among the 121 patients followed for 3 years in this study was approximately €13 000 000. HPV-associated OPC represented 79% of the total cost, and 71% of these cases occurred in males.

## Discussion

This study is the first to use an accurate, bottom-up analysis to describe direct medical costs and indirect costs of OPC stratified by HPV status, cancer stage, and subsite. Our main findings show a mean total cost per patient of €103 386 for HPV-positive and €120 244 for HPV-negative OPC, and that HPV-positive cases represent 79% of the total cost of OPC. Our observations are important for future decisions regarding the implementation of health care strategies for OPC; for instance, whether or not to include males in HPV vaccination programs.

In a recent study, we estimated the annual cost from 2006 of all HPV-associated precancers and cancers in Sweden at €94 million, of which 13% represented costs of OPC [9]. That study used a prevalence-based cost-of-illness design with a top-down approach, using information from the literature on the prevalence of HPV-attributable diseases and data from national registries on costs. The national registries contained data on all patient episodes and their costs, but lacked individual-level information, such as HPV status, cancer stage, etc. For males, OPC represented the majority of the economic burden of HPV-associated cancers, accounting for 56% of the annual societal cost, followed by anal (27%) and penile (17%) cancer. For males, the total annual societal cost of OPC was estimated at €8.5 million, and the corresponding figure for females was €3.4 million. The greatest cost driver for OPC was morbidity and premature death, which represented 78% of the total annual cost.

In contrast to our previous report [9], the present study included patient-specific information from medical records, including HPV status, cancer stage, and OPC subsite, as well as information on disease-specific sick leave and survival. The mean total cost of OPC for the period covering 1 month before diagnosis to 3 years after treatment completion was €106 590 (at the 2017 year’s price level) regardless of gender and HPV status; direct medical costs represented €47 020 (44%) and indirect costs €59 570 (56%) of this total. The mean total cost of HPV-positive OPC was €103 386; the cost of HPV-negative OPC was 16.3% greater (€120 244). The mean total cost for male patients was less (€93 954) than for female patients (€138 925). The difference in costs between men and women in this study reflects the fact that the three women who died during follow-up were relatively young and, therefore, produced high mortality costs. While the influence of HPV on the costs of OPC and the distribution of costs between genders was similar to that reported in our previous study, the present study design allowed us to describe the costs of OPC in a much more specific manner, and also to describe direct medical and indirect costs accurately. Such accurate estimates of the societal cost of OPC, particularly in the context of HPV, are imperative when decisions are being made about the implementation of health care measures.

A series of studies have focused on the costs of HNC, including OPC. Keeping *et al*. [6] estimated the costs of laryngeal and oral cavity cancer, as well as OPC, in secondary care facilities in the UK. Male patients represented a majority of the costs, which is in agreement with the observations in the present study. However, their data are otherwise not comparable to ours, as they did not include indirect costs or detailed patient-specific costs, e.g., by HPV status, cancer stage etc. Lairson *et al*. [8] used an insurance-based setting in Texas and reported direct medical costs of OPC of $139 749 (approximately €119 000) for the first 2 years after diagnosis. Compared to our observations, that cost is high, despite the fact that indirect costs were not assessed, which suggests differences between the two health care systems. Chesson *et al*. [5] estimated the cost of prevention and treatment of HPV-associated OPC to be $43 200 (approximately €36 900) per patient in their specific setting (USA), but indirect costs were not included. Jit *et al*. [16] focused on modelling the cost-effectiveness of two HPV vaccines and produced a lifetime cost estimate for HNC treatment of £15 000 (approximately €16 800), but no indirect costs were included. Kim *et al*. [7] used a top-down perspective to estimate direct medical costs and focused on patients who received surgical treatment as part of their care. This was not the case with our patients among whom surgery was reserved for residual or recurrent disease; our patients received only radiotherapy or chemoradiotherapy. Given the variability in the design, scope, and accuracy of previous work and the present study, comparisons should be made with caution. However, the data presented here is, to the best of our knowledge, the first to describe direct and indirect costs of OPC by HPV status, cancer stage, and subsite from a bottom-up perspective.

In this study, a degree of stage-dependency was observed, i.e., greater costs were incurred for patients with more advanced-stage OPC, with the exception of the most advanced stage, where only costs for palliative care occurred (a.k.a. “best supportive care”). This information allows for future cost analyses in relation to disease progression. Specifically, whether or not extended work-up times, which can allow the cancer to progress, alter the stage of the condition at diagnosis and thereby its associated societal cost. This is particularly valid when evaluating so-called “standardized care programs” introduced for the rapid work-up and rapid treatment of HNC, including OPC [17]. The present design, which allowed for a description of costs of OPC by subsite, may also be useful for specific health care assessments, for example in the context of introducing transoral robotic-assisted surgery for resectable tonsillar cancer instead of radiotherapy or chemoradiotherapy. This may affect direct medical costs to some degree, but it may potentially have more effect on indirect costs, as transoral robotic-assisted surgery may produce less long-time morbidity compared to radiotherapy and chemoradiotherapy. Taken together, data on costs by cancer stage and OPC subsite, as provided by this study, are warranted and may be useful for future cost-effectiveness assessments relevant to OPC.

The strengths of the present study are the accuracy of the individual patient data used to determine HPV status, cancer stage, and OPC subsite, and the fact that information on direct medical costs as well as indirect costs are covered in the databases and were calculated in this report. Furthermore, the curation of clinical data was carried out by two experienced head and neck surgeons (JS and LG). Moreover, all patients were treated at one hospital, i.e., Skåne University Hospital, and represent consecutive cases from the Southern Health Care Region of Sweden. Due to a lack of data, costs arising from informal care carried out by friends and/or family members, transportation, and on health-related quality of life, were not included in our cost estimates. For example, indirect costs of cancer stage IVC were not retrievable using the methods in this study, because these individuals were taken care of by family members. Furthermore, we did not specifically identify the different items generating the direct medical costs, although long-period hospitalization associated with age and a need for surgical treatment of residual or recurrent disease, respectively, may be factors contributing to high direct medical costs.

In conclusion, a cost-of-illness analysis estimates disease-specific costs and ideally provides information on the maximum public savings obtainable if a disease were to be eradicated. The present study identifies the cost of HPV-positive OPC, i.e., cases that may be prevented by HPV vaccination, and we demonstrate that HPV-positive OPC represents 81% of all OPC cases and 79% of their societal cost. Australia, Canada, the USA, and more recently, Norway and the United Kingdom, have all implemented gender-neutral HPV vaccination, which may aid in breaking the rapid increase in the incidence of HPV-positive OPC. The results of this study can be used in future analytical models to aid decision makers in recognizing the potential value of a gender-neutral HPV program in Sweden, which may prevent a further rapid increase of OPC in the country.

## References

[1] Torre LA, Bray F, Siegel RL, Ferlay J, Lortet-Tieulent J, Jemal A. Global cancer statistics, 2012. CA Cancer J Clin. 2015;65: 87–108. 10.3322/caac.21262 25651787

[2] Perez-Ordonez B, Beauchemin M, Jordan RC. Molecular biology of squamous cell carcinoma of the head and neck. J Clin Pathol. 2006;59: 445–453. 10.1136/jcp.2003.007641 16644882 PMC1860277

[3] Stenmark MH, Shumway D, Guo C, Vainshtein J, Mierzwa M, Jagsi R, et al Influence of human papillomavirus on the clinical presentation of oropharyngeal carcinoma in the United States. Laryngoscope. 2017;127: 2270–2278. 10.1002/lary.26566 28304083 PMC6615732

[4] Wang MB, Liu IY, Gornbein JA, Nguyen CT. HPV-positive oropharyngeal carcinoma: a systematic review of treatment and prognosis. Otolaryngol Head Neck Surg. 2015; 153:758–769. 10.1177/0194599815592157 26124261

[5] Chesson HW, Ekwueme DU, Saraiya M, Watson M, Lowy DR, Markowitz LE. Estimates of the annual direct medical costs of the prevention and treatment of disease associated with human papillomavirus in the United States. Vaccine. 2012;30: 6016–6019. 10.1016/j.vaccine.2012.07.056 22867718 PMC6629018

[6] Keeping ST, Tempest MJ, Stephens SJ, Carroll SM, Simcock R, Jones TM, et al The cost of oropharyngeal cancer in England: a retrospective hospital data analysis. Clin Otolaryngol. 2018; 43:223–229. 10.1111/coa.12944 28734109

[7] Kim K, Amonkar MM, Hogberg D, Kasteng F. Economic burden of resected squamous cell carcinoma of the head and neck in an incident cohort of patients in the UK. Head Neck Oncol. 2011;3: 47 10.1186/1758-3284-3-47 22035422 PMC3219567

[8] Lairson DR, Wu CF, Chan W, Dahlstrom KR, Tam S, Sturgis EM. Medical care cost of oropharyngeal cancer among Texas patients. Cancer Epidemiol, Biomarkers Prev. 2017; 26:1443–1449. 28838945 10.1158/1055-9965.EPI-17-0220 PMC5903433

[9] Östensson E, Silfverschiold M, Greiff L, Asciutto C, Wennerberg J, Lydryp ML, et al The economic burden of human papillomavirus-related precancers and cancers in Sweden. PloS One. 2017;12: e0179520 10.1371/journal.pone.0179520 28651012 PMC5484479

[10] Rice DP. Estimating the cost of illness. Am J Public Health Nations Health. 1967;57: 424–40. 10.2105/ajph.57.3.424 6066903 PMC1227175

[11] Cancercentrum [Cited 8 March 2017]. In: Svenskt kvalitetsregister för huvud- och halscancer. Available from: www.cancercentrum.se/samverkan/cancerdiagnoser/huvud-och-hals/kvalitetsregister.

[12] Gronhoj Larsen C, Gyldenlove M, Jensen DH, Therkildsen MH, Kiss K, Norrild B, et al Correlation between human papillomavirus and p16 overexpression in oropharyngeal tumours: a systematic review. Br J Cancer. 2014;110: 1587–1594. 10.1038/bjc.2014.42 24518594 PMC3960616

[13] Statistiska centralbyrån [Cited 5 June 2017]. In: Lönestrukturstatistik, hela ekonomin. Available from: www.scb.se/hitta-statistik/statistik-efter-amne/arbetsmarknad/loner-och-arbetskostnader/lonestrukturstatistik-hela-ekonomin.

[14] Liljas B. How to calculate indirect costs in economic evaluations. Pharmacoeconomics. 1998;13: 1–7. 10.2165/00019053-199813010-00001 10175982

[15] Gold M. Panel on cost-effectiveness in health and medicine. Med Care. 1996;34: S197–199. 8969326

[16] Jit M, Chapman R, Hughes O, Choi YH. Comparing bivalent and quadrivalent human papillomavirus vaccines: economic evaluation based on transmission model. BMJ. 2011;343: d5775 10.1136/bmj.d5775 21951758 PMC3181234

[17] Jensen AR, Nellemann HM, Overgaard J. Tumor progression in waiting time for radiotherapy in head and neck cancer. Radiother Oncol. 2007;84: 5–10. 10.1016/j.radonc.2007.04.001 17493700

